# Association of geographical distribution of air quality index and type 2 diabetes mellitus in Isfahan, Iran

**DOI:** 10.12669/pjms.312.6762

**Published:** 2015

**Authors:** Azadeh Tahmasebi, Mohammad Mehdi Amin, Parinaz Poursafa, Bijan Iraj, Hamidreza Sadeghiyan, Roya Kelishadi, Babak Sadeghian

**Affiliations:** 1 Azadeh Tahmasebi, Environmental Health Engineering Department, Environment Research Center, Research Institute for Primordial Prevention of Non-communicable Disease, Isfahan University of Medical Sciences, Isfahan, Iran; 2 Mohammad Mehdi Amin, Environmental Health Engineering Department, Environment Research Center, Research Institute for Primordial Prevention of Non-communicable Disease, Isfahan University of Medical Sciences, Isfahan, Iran; 3 Parinaz Poursafa, Environmental Health Engineering Department, Environment Research Center, Research Institute for Primordial Prevention of Non-communicable Disease, Isfahan University of Medical Sciences, Isfahan, Iran; 4 Bijan Iraj, Isfahan Endocrine and Metabolism Research Centre, Isfahan University of Medical Sciences, Isfahan, Iran; 5 Hamidreza Sadeghiyan, Pediatrics Department, Child Growth and Development Research Center, Research Institute for Primary Prevention of Non-communicable Disease, Isfahan University of Medical Sciences, Isfahan, Iran; 6 Roya Kelishadi, Pediatrics Department, Child Growth and Development Research Center, Research Institute for Primary Prevention of Non-communicable Disease, Isfahan University of Medical Sciences, Isfahan, Iran; 7 Babak Sadeghian, Isfahan Department of Environment

**Keywords:** Air pollution, Diabetes mellitus, Geographic distribution

## Abstract

**Objectives:**

Air pollution is a hazardous environmental problem with several adverse health effects including its impact on the development of chronic diseases as diabetes mellitus. This study aimed to investigate the association of geographical distribution of air quality index (AQI) and type 2 diabetes mellitus in an air-polluted city by using geographic information system (GIS).

**Methods:**

This cross-sectional study was conducted in Isfahan, Iran. The records that have been registered from 2009 to 2012 in major referral public diabetes clinics were gathered; they included data of 1467 diabetic patients. Their living area was represented with spots in the city map. AQI data were also interpolated from monitoring stations spreading around the city. The GIS maps of air pollutants and diabetes were developed and the associations were determined.

**Results:**

The density of diabetic population was higher in highly polluted areas compared with areas with the lower levels of air pollution. No significant correlation was documented between the distribution of diabetic patients and air pollution level throughout the city.

**Conclusion:**

Although the density of diabetic patients was higher in areas with higher air pollution, but the lack of association between AQI and the prevalence of diabetes might be because the air of different parts of the city was highly polluted, and we could not compare the prevalence of diabetes in areas with clean and polluted air.

## INTRODUCTION

Air pollution is a hazardous environmental problem with several adverse health effects including possible impact on chronic diseases as diabetes mellitus. Among the different types of diabetes, type II diabetes mellitus (T2DM) is the most prevalent type, and its prevalence is increasing worldwide.

T2DM has a complex etiology; in addition to genetic predisposition and unhealthy lifestyle habits, the role of environmental factors is becoming more evident. A growing body of evidence showed that exposure to ambient air pollutants is associated with insulin resistance^1^, cardio metabolic risk factors^2^ and T2DM. It is suggested that the escalating trend of non-communicable diseases includingT2DM might be, at least in part, because of exposure to air pollutants. The results from recent studies have indicated a positive correlation between prevalence of T2DM and air pollutants specially the nitrous oxide (NOx) gases.^3^

It is estimated that the present number of 285 million diabetic patients will reach 330 million in 2025, and will surpass 366 million in the year 2030. The situation of Middle Eastern countries is of special concern in this regard; it is estimated that in the near future, the population of these countries will face the highest burden of T2DM worldwide.^2^ Given the high prevalence of diabetes and high levels of air pollutants in this region, it is necessary to conduct more studies in this regard. Even a weak association between air pollution and diabetes mellitus would be important because of widespread exposure to air pollutants and the high prevalence of diabetes mellitus at population level. As a country in the Middle East, Iran is facing both problems of diabetes mellitus and air pollution.

This study aimed to investigate the association of the geographical distribution of air pollutants and diabetes in an air-polluted city by using geographic information system (GIS).

## METHODS

### Study area and population

Isfahan is an industrial city located in 32°38’30” N latitude and 51°39’40” E longitude with a population of near 1.9 million, located in the center of Iranian plateau, with an average altitude of 1500m from the sea level bounded by NW-SE mountain range of 3000m. Isfahan has very regular seasons due to the mild climate. The average monthly temperature is 16°C with a maximum of 29°C in July and minimum 3°C in December with mild winds from west and south. The air of this city is predominantly affected by industrial emissions and motor traffic, which lead to increasing air pollutant concentrations during stagnant conditions.^4^ This city is ranked as one of the top 10 air-polluted cities of the world.

### Case ascertainment

We defined incident cases of diabetes mellitus as self-report of doctor-diagnosed that have been visited in the main public referral centers providing health care services for diabetic patients. These centers were affiliated to Isfahan University of Medical Sciences and the public insurance clinics. The records registered from 2009 to 2012 including data of 1467 diabetic patients were studied. Their living area was represented with spots in the city map.

### Air pollutants

Data on air quality index (AQI) were obtained from 7 monitoring stations located in different parts of the city. Data from pollution monitoring stations in outspread spots throughout the city was used. The average number of the data from each station recorded between 2009-2012 was digitized on a map by specific information. The maps of diabetes distribution and AQI were prepared by ARC GIS software. Data were interpreted using Inverse Distance Weighting (IDW).

### Statistical analysis

Logistic regression analyses, considering two different adjusted strategies, were conducted to examine the relationship between AQI and diabetes mellitus prevalence in each area. The distribution of spots on the map was assessed by Pseudo R square and Relative Operating Characteristics. In the regression modeling, diabetes prevalence was considered as dependent variable and AQI as independent variable. In addition, regression(R) and determination coefficients (R2; 40.7) were verified and estimate standard, as well as total errors were calculated. P values < 0.05 were considered as statistically significant. Statistical analyses were performed with SPSS software version 20.0 (IBM, Armonk, NY, USA).

## RESULTS

**Fig.1:**
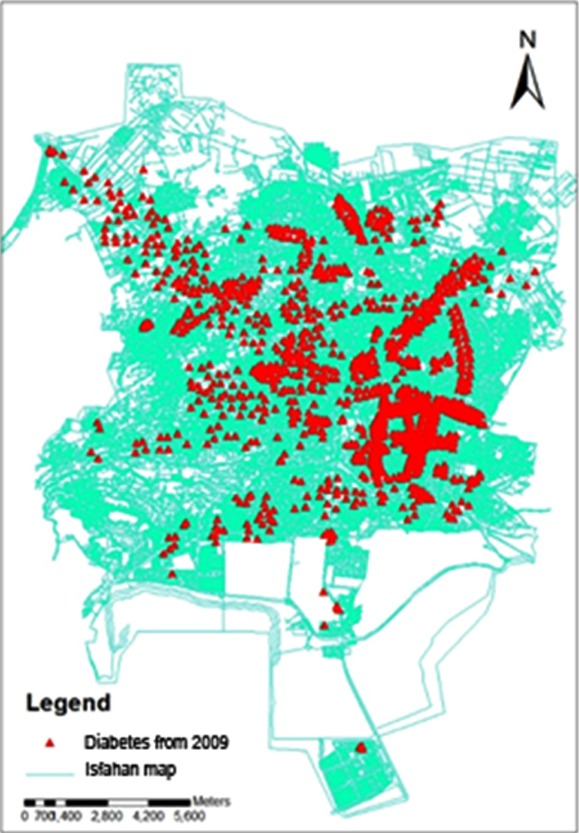
Presence spots of diabetic patients in Isfahan.

**Fig.2:**
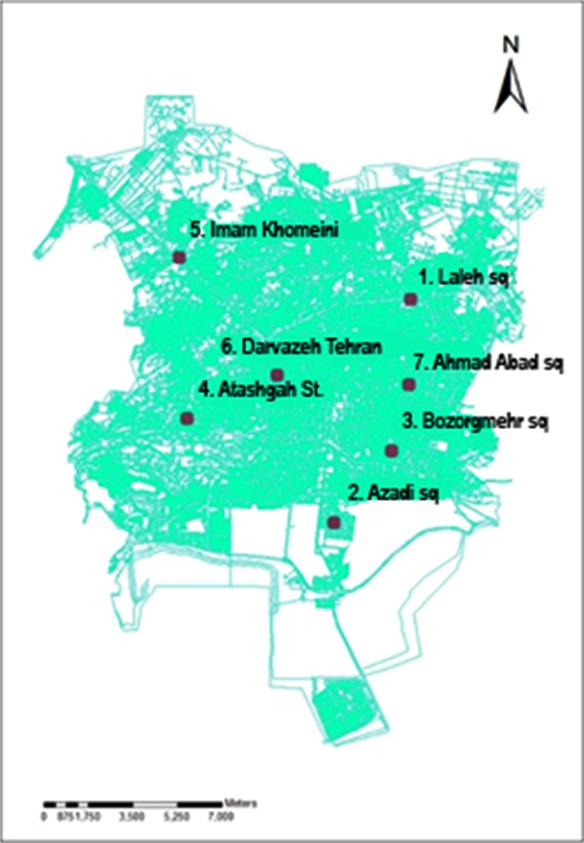
Air pollution measuring stations in Isfahan.

**Fig.3:**
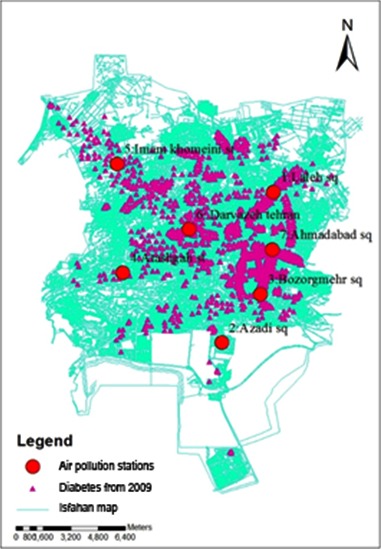
Presence spots of diabetic patients and the air pollution measuring stations.

This study included 1467 adult diabetic patients consisting of 61% female and 39% male individuals. Distribution of diabetes mellitus in the study area is presented in Fig.1. The average number of the AQI data from each monitoring station recorded between the years 2009-2012 was digitized on a map by specific information (Fig.2). The difference between the years of obtaining AQI and diabetes data is because of the lag time in the occurrence of the disease, which was considered at least one year (Fig.3).

**Fig.4:**
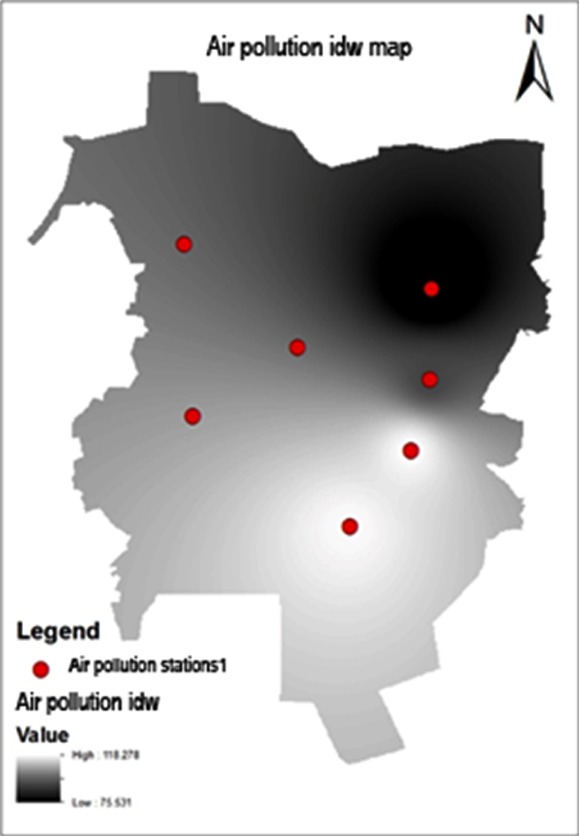
The map of pollution measuring stations weighting.

As depicted in Fig.4, no significant correlation was documented between the distribution of diabetic patients and AQI level throughout the city (R^2^: 0.08). The density of diabetic population was higher in highly polluted area (e.g. Imam-Khomeini and Jomhouri squares) compared with areas with the lowest AQI level (i.e. Laleh square).

## DISCUSSION

In this study, we assessed the relationship of AQI level and diabetes distribution by using their GIS maps. Although the density of diabetes was higher in some areas with high air pollution, in general we did not find significant associations.

To the best of our knowledge, this is the first study of its kind not only in Iran, but also in the Middle East. Our findings are not in line with some other studies that have documented positive relationship between better air quality and lower prevalence of T2DM. Many studies have confirmed significant positive correlations between air pollution based on the distance of patients’ house from the roads and the prevalence of T2DM.^5^-^7^

Controversies exist on the association of air pollutants with diabetes mellitus. Our findings are consistent with some previous studies. A cross-sectional study conducted on 8018 individuals living in a semi-rural area of the Netherlands^8^ did not find association between the air quality and diabetes. Likewise, two cohort studies demonstrated the same findings. A national cohort conducted on 51,818 non-diabetic participants in Denmark showed that during 9.7 years of follow up, ambient NO_2_ level was not related to the incidence of diabetes.^9^ The United States cohort study on 74412 participants revealed similar results.^10^ In the 23- year cohort of Nurse’s Health study and Health Professional follow up, a weak association was found between PM levels and diabetes. However, this association was stronger when analysis was restricted to the final two years of the follow up. This finding might be because of the cumulative effect of exposure to air pollutants over time, and it supports the hypothesis that longer exposure to environmental pollutants has stronger adverse health effects.^10^

On the other hand, some studies have revealed significant associations between air pollutants and diabetes.^2^,^3^,^11^ However, in most of them, no obvious distinction was documented between two types of diabetes, and the overall prevalence was described. A cohort study on 57052 diabetic patients in Denmark documented significant correlation between air pollution from vehicles, mainly the NO2 gas, and the prevalence of T2DMespecially in women.^9^ Likewise in a survey in two different areas of Canada, a higher risk of T2DM was documented in women living in more polluted areas than in their other counterparts. Higher vulnerability to air pollution in women could be because of different physiological inflammatory responses between genders and the differences in life style habits.^12^

A study in the US revealed that the prevalence of T2DM was higher in areas with air pollutants more than standard levels, and even in areas with standard levels of air pollution, those parts with higher levels of pollutants had a higher prevalence of T2DM.^3^

A cohort study in Canada revealed that NO_2_ might increase the odds ratio for the prevalence of diabetes mellitus.^13^ A study in the United States demonstrated that the prevalence of diabetes is particularly associated with levels of ambient particular matter (PM).^14^

Many animal and human studies demonstrated air pollution exposure might be associated with chronic inflammatory conditions including atherosclerosis, increased fasting glucose, and elevated blood pressure.^3^ Findings of a cohort study in Los Angeles, USA revealed that the accumulative adverse health effects during long-term exposure to air pollutants might lead to chronic diseases.^15^ PM might have a role in endothelial dysfunction and might induce inflammation in different parts including in visceral adipose tissue. It also promotes hepatic insulin resistance, mitochondrial dysfunction and brown adipose tissue alteration.^2^ A recent meta analysis revealed the association between PM air pollutants and diabetes mellitus was weaker than that of gaseous pollutants. However, this meta analysis confirmed a weak and significant association between air pollution and diabetes mellitus.^16^

The non-significant findings of the current study might be because in the area under study, the air quality was low in most days of the year and in all different parts of the city, so the whole area under study had poor air quality and we could not compare the prevalence of diabetes in low and high polluted areas. This suggestion is also supported by the findings of a16-year cohort study in Germany that was conducted.^11^ Moreover, we used cross-sectional data, possibly a longitudinal study with long-term follow up might reach significant associations between low air quality and diabetes mellitus.

These differences in results of various studies are due to numerous differences including population characteristics, individual susceptibility, technical aspect of exposure assessment methodologies, air pollutants measurements, pollution types, the duration of exposure and air pollutant particles and size. Sex predilection may be an important factor that can impact the results. Men tend more to be mobile than women and therefore expose more to air pollutant and show consequences of exposure. The levels of ambient particles in outdoor and indoor air should be controlled at source, whether by transport policies or regulatory changes, as well as by increasing public awareness. We have to consider Air pollution more seriously as a novel and modifiable risk factor.

### Study limitations

The most important limitations of this study are the cross-sectional nature of data and the lack of precise exposure to ambient air pollutants. Many people residency is different from their workplaces. It may changes the rate of air pollution exposure. On the other hand, ambient pollution concentrations may not accurately reflect individual exposure. The other limitation we should notice is that we did not distinct new cases of diabetes mellitus from known cases. Moreover we studied the records of the public clinics and not the private ones; however the data used were from the main referral clinics of the city.

## CONCLUSION

We did not find significant association between air pollution level and prevalence of diabetes mellitus; this might be because the air of different parts of the city was highly polluted, and we could not compare the prevalence of diabetes in low- and high-polluted areas. However, air pollution continues to have an adverse effect on the public’s health. It is necessary to reexamine environmental health policies and standards in developing countries.
